# Influence of demographic factors on prolonged length of stay in an emergency department

**DOI:** 10.1371/journal.pone.0298598

**Published:** 2024-03-18

**Authors:** Afnan Alnahari, Ashraf A’aqoulah

**Affiliations:** 1 Public Health Operation Center, Public Health Agency, Ministry of Health, Jeddah, Saudi Arabia; 2 Department of Public Health, College of Public Health and Health Informatics, King Saud bin Abdulaziz University for Health Sciences, Riyadh, Saudi Arabia; 3 King Abdullah International Medical Research Centre, Riyadh, Saudi Arabia; Azienda Ospedaliero Universitaria Careggi, ITALY

## Abstract

**Background:**

A prolonged length of stay in an emergency department is related to lower quality of care and adverse outcomes, which are often linked with overcrowding.

**Objective:**

Examine the influence of demographic factors on prolonged length of stay in the emergency department.

**Methods:**

This study used a cross-sectional design. It used secondary data for all patients admitted during the specific duration at the emergency department of a governmental hospital in Saudi Arabia. The independent variables were gender, age, disposition status, shift time, and clinical acuity (CTAS) level while the dependent variable was prolonged length of stay.

**Results:**

The study shows that 30% of patients stay at the emergency department for four hours or more. The results also show a significant association between demographic factors which are age, gender, disposition status, shift time, clinical acuity (CTAS) level and prolonged length of stay in an emergency department. Based on the results males are more likely to stay in the emergency department than females (OR = 1.20; 95% CI = 1.04 to 1.38). Patients aged 60 and older are less likely to stay in the emergency department than patients aged 29 or smaller (OR = 0.58; 95% CI = 0.39 to 0.84). According to disposition status discharged patients after examination stays in the emergency department more than admitted patients after the examination (OR = 2.78; 95% CI = 1.67 to 4.99). Patients who come to the night shift are less likely to stay in the emergency department than patients who come in the morning shift (OR = 0.67; 95% CI = 0.56 to 0.81). Patients who are classified in level three of CTAS are less likely to stay in the emergency department than patients who are classified in level one (OR = 0.28; 95% CI = 0.88 to 0.023).

**Conclusion:**

Demographic factors such as age, gender, shift time, disposition status and clinical acuity (CTAS) were important factors that needed to be considered to reduce the length of stay of patients in the emergency department. it is possible to formulate a machine learning model to predict the anticipated length of stay in the hospital for each patient. This prediction with an accepted margin of uncertainty will help the clinicians to communicate the evidence-based anticipated length of stay with the patient’s caregivers. In addition, hospital managers need to provide the emergency department with enough staff and materials to reduce the length of stay of patients.

## Introduction

Emergency departments (EDs) in hospitals offer a critical public service and provide emergency medical services at any time [1]. Timeliness is the key to providing prompt treatment and quality of care in a busy ED environment. Therefore, timeliness is particularly measured by “length of stay” in ED. [2] the overcrowding problem in the ED has received a great deal of focus in recent years in many countries [3]. It illustrates the gap between identified emergency care demands and available services in the ED and hospital. Moreover, ED overcrowding has long been recognized as a chronic health problem around the world [4]. Staying for an extended time and the length of stay (LOS) in the ED is used as a critical determinant. A prospective cohort study was conducted in a Dutch tertiary care hospital and included 1434 ED patients. They reported that presenting complaints (number and type), consultation and imaging/laboratory testing time, and ICU admission as the main cause of prolonged LOS-ED. Whereas, if the decision-making time and discharge procedure are accelerated, the LOS-ED will be reduced [5]. Similarly, reduction of LOS may help to lessen ED overcrowding. Studies also reported that periods of increased ED crowding were related to high inpatient mortality with a moderate increase in LOS and costs for hospitalized patients [6].

Numerous characteristics of ED and patients have been related to prolonged ED-LOS. A systematic review of 35 studies showed that previously published studies were insufficient to decide which determinants need to be targeted and modified to improve ED logistics [7]. They demonstrated that most of the studies included retrospective data, whereas concurrent measures are necessary to study time delays in ED. Moreover, Earlier studies regarding longer LOS-ED failed to account for potential influencing factors, such as patients’ gender, age, and admission time. A study was conducted in the Netherlands and included 94 ED nurse managers. They reported that besides the relatively short LOS-ED, 68% of them reported crowding as the main frequent and nationwide problem and occurred many times a week or sometimes daily. This ED crowding has resulted from delays in consultation, laboratory, and radiology testing delays and a shortage of hospital beds for patients requiring admission [8]. To reduce the crowding and restrict the work-up time in EDs in the UK and Netherlands, the “4-Hour rule” was introduced [9]. In an Australian study, they found that after the implication of the 4-h rule the in-hospital mortality rate among the ED admissions declined with an increase in the compliance rate (proportion of patients admitted or discharged from EDs) [10] The LOS in ED is different in countries. In the Netherlands, for discharged patients, the LOS is 119mins and for admitted patients is 146 min [8]. However, in the USA, during times of overcrowding, the LOS for admitted patients extends for up to 24h [11]. Other factors that contributed to the LOS-ED and crowding include shortages in emergency department space or beds or acute care hospital beds, a shortage of nursing staff.

The LOS-ED is challenging for ED management, understanding its main contributing factor, reporting, and analyzing these determinants is important for dealing with this problem [2]. A prolonged LOS-ED is related to lower quality of care and adverse outcome, which is often linked with overcrowding [12]. Similarly, prolonged LOS leads to dissatisfaction of the patients, increases the number of patients leaving the hospital before doctor consultation. Likewise, prolonged LOS-ED of patients leads to unnecessary bed occupation, consumes more time from the staff, and delays the admission of new patients [13]. As a result of this growing challenge, emergency activities can be curtailed or become dysfunctional. There is evidence in the literature that ED crowding may have significant negative implications, such as longer wait times, more complications, and decreased satisfaction, and more patient mortality [14]. Reducing LOS duration is crucial not only to improve ED healthcare but also to reduce healthcare costs. Planning of interventions aimed at reducing time spent in ED needs a proper understanding of its determinants in Saudi Arabia, including demographics of patients and time of admission that can predict the likelihood of its occurrence.

In Saudi Arabia, the growing population has resulted in a rise in ED admissions [15]. As a consequence, improving ED efficiency is a pressing concern. Although various studies have looked into the factors that lead to prolonged ED duration of stay LOS, the bulk of data comes from datasets in developed countries such as the United States and China [16, 17]. These findings do not adequately reflect the situation in countries with different health systems, such as Saudi Arabia. As a result, elucidating the variables associated with ED LOS is necessary to minimize crowding and enhance care delivery in a country with a different health system and population dynamics.

Reduction in LOS has been regarded as a potential strategy to optimize resource consumption and reduce health care costs. Planning of interventions to reduce time spent in the hospital requires understanding organizational and individual factors that may affect LOS and the identification of subgroups of patients with a longer hospital stay. As a result, this study aims to examine the influence of demographic factors on prolonged LOS in the ED.

## Methods

### Study design and setting

A retrospective cross-sectional study was conducted for data collection. The study used secondary data for all patients admitted to a tertiary hospital in Jeddah, Saudi Arabia. Jeddah is one of the largest cities situated in western Saudi Arabia, with an estimated over 3.5 million population. The hospital is considered one of the five largest Ministry of Health hospitals in Jeddah city, serving the community. The hospital has a total capacity of 300 beds and the ED capacity is 36 beds. In addition, all laboratory and radiological examinations are performed in the emergency department [18].

### Study variables

The demographic factors (independent variables) were gender, age, disposition status (admission required, canceled, discharge, referral, left without being seen), shift time (morning, evening, night), and clinical acuity based on the Canadian triage and acuity scale (CTAS) level. The Canadian triage and acuity scale **(**CTAS) was based on the triage categories assigned to patients by the responsible nurses in the triage room. The studied ED uses a 5-level scale. These levels were level 1(Resuscitation- Conditions that are threats to life or limb); level 2 (Emergent–Conditions that are a potential threat to life, limb, or function); level 3 (Urgent–Serious conditions that require emergency intervention); level 4 (Less urgent–Conditions that relate to patient distress or potential complications that would benefit from intervention); level 5 (Non-urgent–Conditions that are non-urgent or that may be part of a chronic problem). While the dependent variable was prolonged length of stay in emergency department (more than or equal to 4 hours; less than 4 hours). Prolonged length of stay is defined as the time from registration to exit that is the time when the patient is admitted to an ED bed, and then transferred or admitted to the hospital bed or to another department, or referred to a different hospital, or discharge from the hospital. These factors were selected because they are documented in EDs. The LOS-ED was a continuous variable measured in minutes, whereas all demographic factors were categorical variables.

### Data collection

All patients who were admitted to the ED during a specific period were included in this study. All data were fully anonymized before accessing the data by authors. The authors accessed the data for research purposes on October 1, 2021. The authors had no access to information that could identify individual participants during or after data collection.

Data were collected from the ED database based on predefined criteria. The medical record of 53874 patients who were admitted to ED was included in the study. All the information related to the patient was documented in the Health Information Management System (HIMS). The data consists of gender, age, disposition status, shift time, and clinical acuity (CTAS) level. Data were extracted from the HIMS and entered into a Microsoft Office Excel sheet of Windows (v14.0). Then all data was transferred to the SPSS program. Data were anonymized. Patients’ names, file numbers, and contact details were not collected.

### Ethical approval

The study was conducted according to the guidelines of the Declaration of Helsinki and approved by the Institutional Review Board (IRB) at King Abdullah International Medical Research Centre (KAIMRC). The study approval number is SP21R/378/06.

### Statistical analysis

Descriptive statistics such as mean, standard deviation, frequency, and percentage were used to describe the demographic factors. Data exploration was done by visualizing the data by making bar charts for categorical variables (Length of stay). Chi-square was also used to find the p-value. Finally, binary logistic regression was used to compute the adjusted odds ratio for the relationship of different variables gender, age, disposition status, shifts time and clinical acuity (CTAS) with a length of stay (outcome variable). The outcome variable was computed by dichotomizing the length of stay (more than or equal to 4 hours; less than 4 hours).

## Results

Table 1 shows the number and percent of demographic factors and length of stay. A total of 6,258 patient visits to the emergency department. According to the gender distribution in these patients was almost equally distributed as 44% were females while 56% were males. Most of the patients were less than 30 years old, they consisted of 65%. Triage was done on patients. Almost all the patients 96.0% were discharged. Distribution of the patients across shifts was almost equal as 39% of the patients presented on the morning, shift, 29% of patients presented on the morning shift, and 32% of the patients presented on the night shift. Based on clinical Acuity (CTAS), the majority of patients were classified under level 4 (77%). The length of stay (outcome variable) shows that 30% of the patients waited in the emergency department for more than or equal to 4 hours.

**Table 1:** Number and percent of demographic factors and length of stay.

Characteristic	N = 6258 N (%)
**Gender**	
Female	2,738 (44%)
Male	3,509 (56%)
Un-Specified	11 (0.2%)
**Age**	
> 30 years old	4054 (65%)
From 30 to 39 years old	996 (16%)
From 40 to 49 years old	528 (8%)
From 50 to 59 years old	367 (6%)
≥ 60 years old	305 (5%)
**Disposition Status**	
Admission Required	216 (3.5%)
Canceled	39 (0.6%)
Discharge	6,001 (96%)
Left without being seen (LWBS)	1 (<0.1%)
Refer	1 (<0.1%)
**Shift Time**	
Evening	1,833 (29%)
Morning	2,449 (39%)
Night	1,976 (32%)
**Clinical Acuity (CTAS)**	
Level 1 (Resuscitation)	15 (0.3%)
Level 2 (Emergent)	33 (0.8%)
Level 3 (Urgent)	465 (11%)
Level 4 (Less urgent)	3,353 (77%)
Level 5 (Non-urgent)	502 (11%)
**Length of Stay**	
≥ 4 hours	1,874 (30%)
> 4 hours	4,384 (70%)

Table 2 shows the relationship between demographic factors and length of stay. The demographic factors have a statistically significant relationship with the length of stay. The demographic factors were gender (P = <0.006), age (P = 0.019), disposition status (P = <0.001), shifts (P = <0.001), and clinical acuity (CTAS) (P = <0.001).

**Table 2:** The relationship between demographic factors and length of stay.

Variable	N	≥ 4 hours, N = 1,064	> 4 hours, N = 3,287	p-value
**Gender**	4,351			0.006
Female		433 (22.4%)	1,496 (77.6%)	
Male		631 (26%)	1,791 (74%)	
**Age**	4,351			0.019
> 30 years old		728 (24.3%)	2,269 (75.7%)	
From 30 to 39 years old		167 (27.9%)	431 (72.1%)	
From 40 to 49 years old		75 (23.4%)	245 (76.6%)	
From 50 to 59 years old		59 (26.3%)	165 (73.7%)	
≥ 60 years old		35 (16.5%)	177 (83.5%)	
**Disposition Status**	4,351			<0.001
Admission Required		15 (9.8%)	138 (90.2%)	
Discharge		1,049 (25%)	3,149 (75%)	
**Shifts**	4,351			<0.001
Evening		334 (26.6%)	924 (73.4%)	
Morning		493 (27%)	1,338 (73%)	
Night		237 (18.8%)	1,025 (81.2%)	
**Clinical Acuity (CTAS)**	4,351			<0.001
Level 1 (Resuscitation)		6 (40%)	9 (60%)	
Level 2 (Emergent)		8 (24.2%)	25 (75.8%)	
Level 3 (Urgent)		78 (16.9%)	384 (83.1%)	
Level 4 (Less urgent)		821 (24.6%)	2,519 (75.4%)	
Level 5 (Non-urgent)		151 (30.1%)	350 (69.1%)	

P-value significate at level p ≤ 0.05

Table 3 shows the logistic regression between demographic factors and length of stay. The results show an association between demographic variables and the length of stay. Based on the results males are more likely to stay in the emergency department than females (OR = 1.20; 95% CI = 1.04 to 1.38). Patients aged 60 and older are less likely to stay in the emergency department than patients aged 29 or smaller (OR = 0.58; 95% CI = 0.39 to 0.84). According to disposition status discharged patients after examination stays in the emergency department more than admitted patients after the examination (OR = 2.78; 95% CI = 1.67 to 4.99). Patients who come to the night shift are less likely to stay in the emergency department than patients who come in the morning shift (OR = 0.67; 95% CI = 0.56 to 0.81). Patients who are classified in level three of CTAS are less likely to stay in the emergency department than patients who are classified in level one (OR = 0.28; 95% CI = 0.88 to 0.023).

**Table 3:** The results of logistic regression showing the variables associated length of stay specified by the cut-off value 4 hours.

Variable		Unadjusted				Adjusted		
	OR	% 95 CI		P-value	OR	% 95 CI		P-value
		Lower	Upper			Lower	Upper	
**Gender**								
Female (RC)	1				1			
Male	1.22	1.06	1.40	0.006	1.20	1.04	1.38	0.012
**Age**								
> 30 years old (RC)	1				1			
From 30 to 39 years old	1.21	0.99	1.47	0.061	1.11	0.91	1.36	0.3
From 40 to 49 years old	0.95	0.72	1.25	0.7	0.91	0.68	1.19	0.5
From 50 to 59 years old	1.11	0.81	1.51	0.5	1.03	0.75	1.40	0.9
≥ 60 years old	0.62	0.42	0.88	0.011	0.58	0.39	0.84	0.005
**Disposition status**								
Admission required (RC)	1				1			
Discharge	3.06	1.85	5.47	<0.001	2.78	1.67	4.99	<0.001
**Shifts**								
Evening (RC)	1				1			
Morning	1.02	0.87	1.20	0.8	1.05	0.89	1.24	0.5
Night	0.64	0.53	0.77	<0.001	0.67	0.56	0.81	<0.001
**Clinical Acuity (CTAS)**								
Level 1 (RC)	1				1			
Level 2	0.48	0.13	1.81	0.3	0.45	0.12	1.77	0.2
Level 3	0.30	0.11	0.93	0.028	0.28	0.09	0.88	0.023
Level 4	0.49	0.18	1.46	0.2	0.41	0.14	1.28	0.11
Level 5	0.65	0.23	1.96	0.4	0.55	0.19	1.74	0.3

P-value significate at level p ≤ 0.05; **RC**: Reference Category; **CI**: Confidence Interval

## Discussion

Though the requirements for each hospital’s emergency department are different depending on the patient flow, the catchment area, and many other known and unknown factors, one thing is for sure based on historical data of the hospital records it is important to ensure optimal planning for the hospital’s emergency department’s scheduling system to ensure the provision of quality care to the patients. Increased length of stay is not only a factor that can badly affect only patients’ perceived quality of care, rather it can influence some important aspects of healthcare delivery and can increase morbidity and mortality of the patients. The problem seems obvious, but the solution mostly is not straightforward. Mostly ensuring a smooth provision of services to the patients presenting to the emergency department is dependent on several factors [19]. The complexity of managing this problem is added by the fact that there are different rules which play a role in the overcrowding of the patients in the emergency department.

The study found that shift time, disposition status, clinical acuity (CTAS), gender and age are important factors that are related to the prolonged length of stay in the emergency department. The relationship between shift time and the length of stay is consistent with the findings of other similar studies [20]. There is a fact that there is a difference between the availability of human resources on the morning shift versus the evening or night shift, during the weekdays versus weekends, or different vacations. Most hospitals lack an evidence-based approach to project the demand of possible emergencies and therefore, most hospitals end up becoming crowded, more in some shifts as compared to others. Additionally, there is informal coordination among the staff, and some procedures are followed which are not documented [21]. Same is the conclusion from a study done in Canada which also reported that length of stay is related to the shift in which patient arrives [22]. One important reason that has been reported in other studies is that doctors will tend to keep the patient admitted till morning for further workup [23]. To ensure uniformity in the length of stay across different shifts there is a need to adopt an appropriate scheduling algorithm to optimize the length of stay as it was demonstrated in a hospital in West China [24]. Since there are multiple rules and different objectives so in this case study, multi-objectives optimization scenario was created. At the same time, relevant rules or limitations were included in the constraints. It is required that hospital administration take care of over-crowding of some shifts versus others as scheduling problems and plan efficiently to ensure delivery of timely quality services to the patients [25].

Disposition status was also an important factor that was related to the length of stay. Those who require admission are expected to suffer from serious conditions and therefore would require a shorter stay in the emergency department and will be moved to their respective departments and wards as compared to those who were discharged. This is consistent with another study that found that length of stay in the emergency department is an important factor that predicts a person’s duration of stay in the hospital [26]. The reason for this relationship might be that those who remain in the emergency department for a shorter duration might be suffering from serious or complicated illnesses and may be elderly and therefore it is not easy to stabilize them. Even when they are stabilized, they will require inpatient monitoring before they are discharged therefore, they end up being admitted to regular in-patient wards rather than getting discharged directly from the emergency department. This finding has an important implication for hospital administration. Many times, the hospital’s emergency department is crowded because patients who enter the hospital through emergency are required to be admitted in different wards but since beds are not available in the relevant ward, therefore, beds at the emergency department also remain occupied. It is therefore important that hospital administration re-design the resources in a way so that the nonavailability of beds in the regular wards does not end up crowding the emergency department. It is also needed to be remembered that a hospital is a system of continuum of care for the patient rather than an entity that is divided between different wards and units.

Clinical acuity (CTAS) has a statistically significant relationship with the length of stay in the final model. This finding is in discordance with the findings reported in the published literature [22, 27]. The reason for this finding maybe that there are many other important variables that affect the length of stay in the emergency department and we could not adjust for those factors that our analysis. Some of the factors that might have contributed to the unknown confounding effect may include the diagnosis of the patient, the presence of chronic conditions, the language of the patient and his or her caretaker who is coming to the hospital, and many others.

Gender is also an important variable when predicting the length of stay. Gender-based disparities have been reported in the emergency department in other parts of the world as well [28]. One study has even found that there is a gender disparity in terms of the usage of emergency services [29]. The study found that there was a statistically significant relationship between gender and the LOS-ED. Male gender was associated with increased length of stay at emergency department. This might be because males most admission via accident and emergency department. More males tend to be drivers and are more likely to be victims of road accident, hence, emergency admission and increase the LOS-ED than female [30]. The study also revealed that patients aged 60 and older are less likely to stay in the emergency department than patients aged 29 or younger. This could be because old people have worse health status than young people. It could be also that the emergency staff gives priority and more attention to old people than young people as a kind of respect.

The strength of this study is that the study was done on a large patient admission dataset from of the major hospitals in Saudi Arabia. The data was taken from well-organized electronic medical records. Finally, robust statistical analysis done in this study enabled us to draw conclusions from the final model by adjusting the estimates for the covariates.

There were several limitations to this study. First, this study was done on patients from a tertiary hospital, therefore can only be generalized to similar hospitals in Saudi Arabia. Very limited patient-level information related to the patient’s ethnicity, religious practices, country of origin, and distance from which the patient was included in the study. Additionally, the lack of information about the patient’s reason for showing up to the emergency department and the patient’s diagnosis was also an important limitation as it is a very important factor that can affect the length of stay of the patient. Future studies need to focus on the causes that make the patients come to the emergency department.

## Conclusion

This study concludes that gender, age, shift time, disposition status, and acuity scale were important factors that needed to be considered while predicting the length of stay in patients presenting to the emergency department. In the presence of information on these variables, it is possible to formulate a machine learning model to predict the anticipated length of stay in the hospital for each patient. This prediction with an accepted margin of uncertainty will help the clinicians to communicate the evidence-based anticipated length of stay with the patient’s caregivers. Moreover, such a prediction model can be used to optimize the performance of the emergency room by ensuring the provision of the requisite resources including human resources so that each patient gets is given due diligence and personalized care during his or her stay in the emergency department. In addition, hospital managers need to provide the emergency department with enough staff and materials to reduce the length of stay of patients at the emergency department.

## Supporting information

S1 File (XLSX)
